# Containment measures for emerging and re-emerging vector-borne and other infectious diseases of poverty in urban settings: a scoping review

**DOI:** 10.1186/s40249-018-0478-4

**Published:** 2018-09-03

**Authors:** Laurence Campeau, Stéphanie Degroote, Valery Ridde, Mabel Carabali, Kate Zinszer

**Affiliations:** 1 University of Montreal Public Health Research Institute (IRSPUM), Montreal, Quebec, Canada; 2 French Institute For Research on sustainable Development (IRD), IRD-Paris Descartes University (CEPED), Paris Sorbonne Cités University, Erl Inserm Sagesud, Paris, France; 3 McGill University, Montreal, Quebec, Canada

**Keywords:** Vector-borne diseases, Containment measures, Interventions, Urban health, Scoping review

## Abstract

**Background:**

The emergence and re-emergence of vector-borne and other infectious diseases of poverty pose a threat to the health of populations living in urban and low-income settings. A detailed understanding of intervention strategies, including effectiveness of past outbreak containment, is necessary to improve future practices. The objective was to determine what is known about the effectiveness of containment measures for emerging and re-emerging vector-borne and other infectious diseases of poverty in urban settings and identify research gaps and implications for public health practice.

**Main body:**

We conducted a scoping review and systematically searched peer-reviewed and grey literature published between 2000 and 2016. Different data extraction tools were used for data coding and extraction, and data on implementation process and transferability were extracted from all studies. A quality assessment was conducted for each included study.

We screened 205 full-text articles and reports for a total of 31 articles included in the review. The quality of the studies was generally low to moderate. The largest body of evidence concerned control activities for Ebola virus and dengue fever. The majority of interventions (87%) relied on multiple types of measures, which were grouped into four categories: 1) healthcare provision; 2) epidemiological investigation and/or surveillance; 3) environmental or sanitary interventions; and 4) community-based interventions. The quality of the majority of studies (90%) was poor or moderate, and one-third of the studies did not provide a clear description of the outcomes and of the procedures and/or tools used for the intervention.

**Conclusions:**

Our results highlight the difficulty of establishing causation when assessing the effect of containment measures. Studies that extend beyond solely reporting on effectiveness and take into account the complexity of real-world settings are urgently needed. We recommend the allocation of research efforts to the evaluation of the implementation processes of interventions as well as their comprehensive and systematic description using validated checklists.

**Electronic supplementary material:**

The online version of this article (10.1186/s40249-018-0478-4) contains supplementary material, which is available to authorized users.

## Multilingual abstracts

Please see Additional file 1 for translations of the abstract into the five official working languages of the United Nations.

## Background

Almost a year and a half after the declaration of the Zika virus outbreak as a Public Health Emergency of International Concern, public health practitioners and policy-makers remain indecisive about Zika control measures [1]. This raises concerns about the ability of current systems to deal with the unpredictable nature of emerging pathogens, particularly with 84 countries having confirmed local transmission of the disease since 2015 [2]. Although the Zika virus shares similar features with dengue fever and chikungunya, the clinical and public health communities were caught off guard, given the serious consequences of fetal infections and the rapid spread of the disease [3]. As the Zika pandemic will certainly not be the last emerging infectious disease to challenge global health systems, it is necessary to understand the common knowledge gaps in outbreak response across previous epidemics to improve the containment of future outbreaks.

This is particularly important considering the emergence of new challenges for cities, including demographic and environmental changes. With approximately half of the world’s population now living in urban settings and with the rapid population growth occurring in low-income countries, urban and low-resource settings are particularly prone to epidemics [4]. New megacities act as perfect incubators for the introduction of diseases, with the accelerated and often uncontrolled urbanization resulting in amplified circulation of pathogens due to high population densities and mobility, weak infrastructure and waste management services, and poor housing [5].

There is an important absence of evidence to guide effective prevention and control of epidemics in urban and low-resource settings [6, 7]. The objective of this review was to examine research literature on the effectiveness of containment measures for emerging and re-emerging vector-borne and other infectious diseases of poverty in urban settings. A secondary objective was to identify research gaps and research limitations, and their implication for public health practice.

## Methods

### Description of the Delphi process used to select the six topics of the scoping reviews

This study is part of a larger series of six scoping reviews conducted by the “VEctor boRne DiseAses Scoping reviews” (VERDAS) consortium following a call from the Vectors, Environment and Society unit of the Special Programme for Research and Training in Tropical Diseases (TDR) hosted by the World Health Organization (WHO). The integral protocol of the VERDAS consortium has been published [8].

We used an eDelphi survey (a Delphi survey conducted via electronic mail) to select the six topics considered of highest priority by a panel of 84 international expert participants (43% researchers; 52% public health decision-makers; 5% from the private sector). The eDelphi consisted of a three-round process: 1) we invited participants to suggest any topic to be considered; 2) more than 80 topics were rated from “1–eliminate” to “5–top priority”; and 3) the 20 topics rated 4 or 5 by more than 65% of the participants (i.e., the most highly voted items) were rated for a second time. By the end of the third round, six topics had been selected, with the present topic having obtained the mean rate of 4.00 ± 1.02 and being ranked fifth out of six in terms of importance (71.4% of the participants rated the topic 4 or 5).

### Search strategy

We conducted a systematic search through MEDLINE, Embase, Global Health, Web of Science, and the Cochrane Database of Systematic Reviews in July 2016 to identify published studies. The search strategy was validated by a public health librarian and consisted of the following combination of terms: “vector-borne disease*” OR “infectious disease*” AND “urban setting*” AND “epidemic*” AND “containment measure*” AND “evaluat*”. We added all possible word variations and MeSH terms for each database (see full list in Additional file 2). Truncations, wild cards, and proximity operators were also used to broaden our search. Grey literature was identified through OpenGrey, the Grey Literature Report, and WHO Library Information Networks for Knowledge Database (WHOLIS). Finally, additional articles were identified by manually screening the references of papers that met our inclusion criteria.

### Study selection

The literature identified through the search strategy was independently reviewed by three team members (LC, KK, SD) after a pilot round. Based on the initial review, post-hoc inclusion and exclusion criteria (i.e., developed after the pilot round for the subsequent literature selection) were developed, which stipulated the articles must: 1) be written in English or French; 2) be published in national and international peer-reviewed journals or grey literature reports from relevant organizations; 3) pertain to evaluation of the effectiveness of containment measures in an urban context; 4) pertain to an outbreak, an epidemic, or a pandemic; 5) concern emerging or re-emerging vector-borne diseases or infectious diseases of poverty on humans. To ensure a contemporary overview of outbreak control strategies, we also chose to exclude articles concerning endemic diseases and articles published before January 2000.

The articles that met the inclusion criteria after the title and abstract screening by the two reviewers (LC, KK) were then reviewed in full by the same reviewers. A third reviewer (SD) was consulted to resolve any discrepancies at each stage of the process.

To respect the inclusion criteria objectively, we also based our selection of studies on specific definitions. First, vector-borne diseases were defined as a group of pathogens transmitted between hosts through infected arthropod species such as mosquitoes, fleas, ticks, flies, sandflies, triatomine bugs, and certain freshwater aquatic snails [9].

The term ‘infectious diseases of poverty’, rather than designating a specific group of diseases, is used in global health to describe communicable diseases known to disproportionately affect poorer populations [10]. For this reason, we restricted our scope to interventions conducted in low- and middle-income countries as defined by the World Bank [11].

Second, the terms ‘epidemic’ and ‘pandemic’ refer to the occurrence of cases of a specific disease in higher proportions than normally expected in a specific population and area [12]. Those terms refer to national and international events, respectively. The term ‘outbreak’, being less restrictive, refers to both geographical contexts and can also be used in the context of a single case of an emerging or re-emerging disease [13].

Third, we used data from the 2014 revision of the World Urbanization Prospects issued by the Population Division of the Department of Economic and Social Affairs of the United Nations to determine what should be considered urban populations according to criteria set by each specific country [14].

#### Study characteristics, quality assessment, and data extraction

Descriptive characteristics, quality assessment, and data from articles that met the inclusion criteria were extracted into a standardized template using a Microsoft Excel 2016 (Microsoft corporation, Redmond, Washington, USA) spreadsheet that was validated by two contributors (LC, SD), with an agreement of over 85% for the extracted data. First, the quality of the papers was assessed using the Mixed Methods Appraisal Tool (MMAT) [15]. This tool evaluates the methodological validity of qualitative, quantitative, and mixed methods studies. Studies were ranked according to their respect of specific criteria and were labelled ‘yes’, ‘no’, or ‘don’t know’, depending on whether they clearly met the criteria, did not, or if it was not possible to determine from the reporting whether they met them.

Completeness of intervention description was assessed using the Template for Intervention Description and Replication (TIDieR, http://www.equator-network.org/reporting-guidelines/tidier/) checklist developed by Hoffman and colleagues [16]. This tool was used to document the rationale, materials, procedures (how, by whom, when and where intervention took place), modifications, and fidelity of the intervention [17] (see Additional file 3 for the complete extraction grid used for this review).

The Analysis of the transferability and support to adaptation of health promotion interventions (ASTAIRE, https://www.cairn.info/load_pdf.php?ID_ARTICLE=SPUB_146_0783) checklist, developed by Cambon and colleagues, was used to evaluate the transferability of the interventions, i.e., “the extent to which the result of one intervention in a given context can be achieved in another context” [18].

## Results

### Description of included studies

**Fig. 1:**
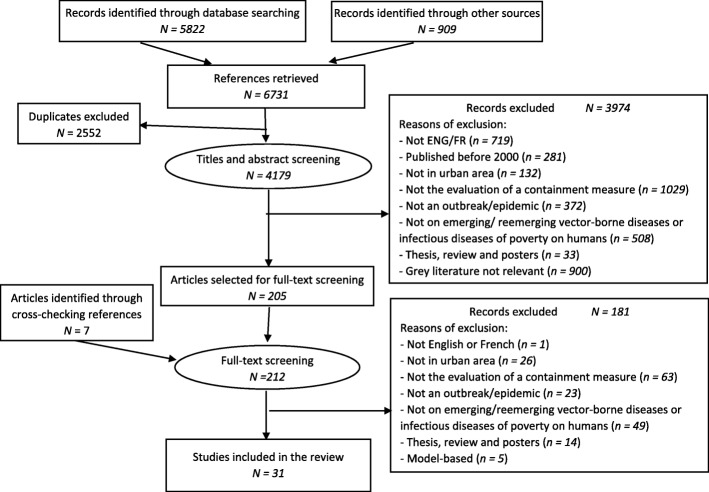
PRISMA Flowchart

Our search strategy yielded 4179 documents in total. The title and abstract screening led to the selection of 205 documents, of which 31 articles met our inclusion criteria after the full-text screening (see the the Preferred Reporting Items for Systematic Reviews and Meta-Analyses [PRISMA] flowchart – Fig. 1). Studies were carried out in Africa (*n =* 14; 45%), South America (*n =* 4; 13%), Asia (*n =* 8; 26%), the Caribbean (*n =* 4; 13%), and Oceania (*n =* 1; 3%). Diseases included Ebola (*n =* 9; 29%), dengue fever (*n =* 7; 23%), cholera (*n =* 5; 16%), Lassa fever (*n =* 2; 6%), A/H1N1 influenza (*n =* 2; 6%), severe acute respiratory disease (*n =* 3; 10%), multi-drug resistant tuberculosis (*n =* 1; 3%), meningitis (*n =* 1; 3%), and malaria (*n =* 1; 3%). Seven (23%) of the studies were mathematical models, two (6%) were observational studies, 12 (39%) were descriptive analyses, and 10 (32%) were case reports. All documents included were peer-reviewed articles published in scientific journals. No documents obtained through the grey literature search met the study criteria.

Characteristics of the included studies are detailed in Table 1. A brief description of the intervention, the outcome measures, an overview of the evaluation of effectiveness, and the main limitations of the study are reported.

**Table 1:** Summary of included studies

Authors, Date	Disease (country)	Types of intervention	Types of measures	Outcome measures	Effectiveness/rapidity	Main limitations
Section I - Case studies						
Santa-Olalla et al. 2013 [19]	Cholera (Haiti)	Alert & response (A&R) system; identification and assessment of cholera alerts and hotspots; organization of a rapid response with partners to provide immediate support based on needs identified in the field (e.g., supplies, training, social mobilization, water, and sanitation)	Healthcare provision; epidemiological investigation and/or surveillance; community-based measures	Not mentioned	Positive (A&R system showed how the rapid detection of cholera alerts was a key element in identifying outbreaks and in directing and coordinating urgent response)	No clear outcome to evaluate the effectiveness of the A&R system
Gazin and Louissaint 2011 [20]	Cholera (Haiti)	Awareness campaign; oral rehydration points; cholera treatment centres; water purification	Healthcare provision; community-based measures; environmental and sanitary interventions	Fatality rate	Positive (mortality rate < 1% in treatment centres; strategy and coordination qualified as ‘efficient’)	Few outcome measures; basic evaluation of effectiveness
Guévart et al. 2005 [21]	Cholera (Cameroon)	Case management; preventive antibiotic treatment of all patient contacts; enhanced surveillance system	Healthcare provision; epidemiological investigation and/or surveillance	Incidence of notified cases from special units	Positive (no new cases after the implementation of containment measures)	The role of large-scale treatment of antibiotic prophylaxis in ending the cholera outbreak could not be ascertained
Bin Yunus et al. 2001 [22]	Dengue (Bangladesh)	Development of national guidelines for clinical management; training of doctors; reorientation of specialists; entomological mapping; documentation of cases; operational studies for testing case definitions; collection of sero-evidence; community-based measures (community empowerment for prevention and control)	Healthcare provision; epidemiological investigation and/or surveillance; community-based measures	No clear outcome	Positive (successful operationalization of action plans)	No clear objective
Maciel-de-Freitas et al. 2014 [23]	Dengue (Brazil)	Standard chemical and environmental vector control measures	Environmental and physical interventions	House index ^a^; number of cases (no information whether laboratory validated); mean incidence	Negative (only slight decrease in vector density; no significant change in seasonal dynamics of dengue)	No clear objective; some highly-productive cryptic containers were not inspected by vector control professionals
Khan and Abbas 2014 [24]	Dengue (Pakistan)	Creation of a provincial cabinet committee; data collection; training of professionals; awareness campaigns; improvement of health infrastructure; appointment of public health officers	Healthcare provision; epidemiological investigation and/or surveillance	Numbers of reported clinical cases	Positive (252 reported cases of dengue in 2012 in Lahore with no deaths)	Limited outcome measures
Nyenswah et al. 2015 [25]	Ebola virus disease (Liberia)	Active case-finding; contact tracing; effective triage within the healthcare system; rapid isolation of symptomatic contacts; home-based and community quarantine; decentralized management of outbreak activities	Epidemiological investigation and/or surveillance; healthcare provision; environmental and sanitary interventions	No outcome measures	Positive (more complete contact tracing, more prompt isolation of symptomatic patients in the second and third generations of transmission, increased survival, and reduced transmission in the community)	Methodology unspecified
Althaus et al. 2015 [26]	Ebola virus disease (Nigeria)	Surveillance and contact tracing; case management; screening of all arrivals/departures in and out of the country by land, air, and sea; social mobilization; use of technology for innovative applications in the EOCs	Healthcare provision; Epidemiological investigation and/or surveillance	Fitting a transmission model to Nigeria outbreaks	Positive	Methodology and outcomes measures not specified
Webster-Kerr et al. 2011 [27]	Malaria (Jamaica)	Early detection and prompt treatment of cases; vector control; public education; community-based surveillance; intersectoral collaboration	Healthcare provision; epidemiological investigation and/or surveillance; community-based measures; environmental and sanitary interventions	Number of laboratory confirmed cases	Positive (only one of 358 cases who had a post-treatment smear on day 7 had a persistent asexual parasitaemia, while none of the 149 persons who had a follow-up smear on day 28 was positive)	No individual assessment of measures’ efficacy; data were observational rather than experimental
Brostrom et al. 2011 [28]	Multi-drug resistant TB (Micronesia)	Case identification; contact investigation; creation of an action plan; construction of MDR-TB isolation units; training of community health workers; enhanced access to national and international subject-matter experts	Healthcare provision; epidemiological investigation and/or surveillance	TB-related mortality rates	Positive (in the 12 months following implementation of these programmatic improvements, TB mortality in Chuuk dropped from 11% of all TB cases to less than 1%)	Limited outcome measures
Section II - Descriptive studies						
Shen and Niu 2012 [29]	A/H1N1 influenza (China)	Screening at borders; isolation; quarantine; large-scale reactive vaccination campaign	Healthcare provision; epidemiological investigation and/or surveillance; environmental and sanitary interventions	Incidence of laboratory confirmed H1N1 influenza; mortality rates	Positive (epidemic curve showed that control measures of containment and vaccination reduced H1N1 morbidity/mortality)	Laboratory confirmed cases analyzed represented a small subset of cases of pandemic H1N1 influenza during the period, as only laboratory-confirmed cases were analyzed
Guévart et al. 2007 [30]	Cholera (Cameroon)	Follow-up of notifications; bacteriological monitoring; antibiotic distribution; large-scale targeted antibiotic prophylaxis	Healthcare provision; epidemiological investigation and/or surveillance	Proportion of contacts among new cases; development of resistant strains	Uncertain (antibiotic prophylaxis limited inter-human transmission of cholera but no impact on the epidemic was shown)	New cases of cholera were not always the consequence of contact with a case, instead resulted from environmental exposure (e.g., contaminated water), such that it was difficult to measure the impact of a preventive strategy at the population level
Peng et al. 2012 [31]	Dengue (China)	Vector surveillance; human surveillance; chemical and environmental vector control measures; community-based measures (prevention, public awareness)	Epidemiological investigation and/or surveillance; community-based measures; environmental and sanitary interventions	Breteau index ^b^; number of dengue cases	Positive (drop of the Breteau index, no more dengue cases reported since September 14, 2010)	No clear objective; only suspected dengue cases were sent for laboratory confirmation
Maciel-de-Freitas et al. 2014 [32]	Dengue (Brazil)	Intensification of standard chemical and environmental vector control measures	Environmental and physical interventions	Infestation levels, number of eggs in ovitraps	Negative (infestation levels only slightly reduced)	Reasons for low efficacy remain unclear due to several uncontrolled variables
Seidahmed et al. 2012 [33]	Dengue (Sudan)	Health education; house inspection campaign by community volunteers; house inspection by health workers; space spraying; larviciding	Healthcare provision; community-based measures; environmental and sanitary interventions	Entomological indices; dengue incidence of laboratory confirmed cases	Both positive and negative, depending on types of measures	No individual assessment of control measure
WHO Ebola Response Team 2016 [34]	Ebola virus disease (Guinea, Liberia, and Sierra Leone)	Case-finding; contact tracing; cases isolation; specially designed Ebola treatment centres; supportive clinical care; safe burials	Healthcare provision; epidemiological investigation and/or surveillance	Does not apply (purely descriptive study)	Positive	Did not explicitly evaluate the impact of the interventions
Abramowitz et al. 2015 [35]	Ebola virus disease (Liberia)	Community-based measures (prevention; training; surveillance; response and treatment; post-outbreak measures)	Community-based measures	Does not apply (qualitative study)	Negative (interventions regarded as necessary, but less desirable than a well-supported health-systems based response; community health messaging failed to provide the needed practical information and training)	Large number of participants in focus groups; questions were posed as hypothetical rather than concerning local experiences and actions
Okware et al. 2015 [36]	Ebola virus disease (Uganda)	Appointment of a national task force; community mobilization; community-based case search, isolation, and public education; improvement of health infrastructures; early detection and action	Healthcare provision; Epidemiological investigation and/or surveillance; community-based measures; environmental and sanitary interventions	Numbers of laboratory confirmed cases; case fatality rate; delays in early detection	Negative in urban settings	Limited outcome measures; methodology not mentioned; did not explicitly evaluate the impact of the interventions
Iroezindu et al. 2015 [37]	Lassa fever (Nigeria)	Contact tracing; risk assessment; decontamination of the environment; establishment of a phone-based alert management system; provision of post-exposure prophylaxis for exposed individuals	Healthcare provision; epidemiological investigation and/or surveillance; environmental and sanitary interventions	Number of secondary cases	Positive (no secondary case of LF occurred)	
Ajayi et al. 2013 [38]	Lassa fever (Nigeria)	Coordination; active surveillance and community mobilization; suspect and contact evaluation; case management	Healthcare provision; epidemiological investigation and/or surveillance; community-based measures	Case fatality rate; timing of outbreak detection	Positive	Basic evaluation of effectiveness
Pang et al. 2003 [39]	Severe acute respiratory syndrome (China)	Set up of fever clinics; training health care workers; closure of all public entertainment sites; construction of designated SARS hospitals with air extraction fans on windows or walls; quarantine of close contacts; self-quarantine	Healthcare provision; environmental and sanitary interventions	Attack rate; number of case; time lag between illness onset and hospitalization	Positive (multiple control measures implemented in Beijing likely led to the rapid resolution of the SARS outbreak)	Could not determine which intervention(s) was the most effective because of the simultaneous and overlapping implementation of multiple control measures; SARS attack rates might have been falsely elevated due to the unavailability of laboratory testing; the 5 districts selected to evaluate contact tracing and quarantine might not have been representative of all of Beijing.
Liang et al. 2005 [40]	Severe acute respiratory syndrome (China)	Infection source control; timely hospital admission and safe transfer of all identified cases; classified isolation of all contacts and suspect cases; centralized treatment and personal protection equipment	Healthcare provision; environmental and sanitary interventions	Interval between disease onset and hospital admission	Positive (notable shortening of the interval between disease onset and notification)	Limited information on control measures; only measured the aggregated effect of multiple intervention measures
Section III - Analytic studies						
Sévère et al. 2016 [41]	Cholera (Haiti)	Reactive vaccination campaign	Healthcare provision	Rates of culture-confirmed cholera, severe dehydration at admission	Positive (only 18 of the 52 357 vaccine recipients (0.034%) had culture-confirmed cholera compared with 370 of the 17 643 unvaccinated (2.09%); no case of cholera had been documented in a vaccine recipient since September 2013).	Study not designed as a case-control study; impact of natural immunity to cholera not taken into account; heterogeneity of risk for cholera within the catchment area; impact of migration; passive surveillance for acute diarrhea cases; probable that asymptomatic or mild cases did not present to the treatment centres; migration of population in and out of the slum may also impact the estimated herd immunity; impact of interventions may be difficult to differentiate.
Ordóñez González et al. 2011 [42]	Dengue (Mexico)	Chemical vector control measures	Environmental and sanitary interventions	Mosquito mortality rates (15 min and 24 h after exposure)	Positive (mosquito mortality rates of 78.8% to 96.6% after 15 min of exposure and 98.8% to 100% 24 h after exposure. No mortalities were observed in the controls)	Small sample size (4 houses with 3 cages in each)
Section IV – Model-based						
Tang et al. 2012 [43]	A/H1N1 influenza (China)	Contact tracing; campus quarantine	Epidemiological investigation and/or surveillance; environmental and sanitary interventions	Peak time of the epidemic; magnitude of outbreak (number of infectious individuals)	Positive (reduction of A/H1N1 transmission from the campuses into the wider community; delay in timing of peak of infection)	Generalization of findings is limited because of unique features of the social network and academic activities in Chinese campuses
Pinho et al. 2010 [44]	Dengue (Brazil)	Adult vector control mechanisms	Environmental and sanitary interventions	Number of cases (no information whether laboratory validated)	Negative (reduction in total number of cases; resurgence of the epidemic process (R(t) > 1) as a consequence of susceptible humans)	Limited information on control measures
Merler et al. 2015 [45]	Ebola virus disease (Liberia)	Deployment of protection kits to households; Ebola treatment units; safe burials	Healthcare provision; environmental and sanitary interventions	Number of projected cases and deaths	Positive (Ebola treatment units may have contributed to halving the number of cases and deaths; deployment of protection kits to about 50% of households may have contributed to further reduce incidence from 30 new cases daily to 10; safer burial practices may have contributed an additional 50% reduction compared to no intervention)	Data availability is limited (some estimates were obtained from previous outbreaks); quantitative assessment of effectiveness and coverage of protection kits was not possible
Althaus et al. 2015 [26]	Ebola virus disease (Nigeria)	Case isolation; contact tracing; surveillance	Epidemiological investigation and/or surveillance; environmental and sanitary interventions	Change in net reproduction number after implementation of control interventions; risk of outbreak from a single undetected case	Positive (reduction of net reproduction number Rt below unity 15 days (95% *CI*: 11–21 days) after the arrival of the index case)	Fitting of a deterministic model to a small outbreak (20 cases); assumption that EVD cases are equally infectious throughout their infectious period; did not examine the separate contributions of transmission in healthcare settings and in the community; did not distinguish between different types of interventions; treated the two transmission clusters as a single outbreak
Kucharski et al. 2015 [46]	Ebola virus disease (Sierra Leona)	Introduction of treatment beds in Ebola holding centres	Healthcare provision	Number of cases averted	Positive (56 600 (95% credible interval: 48300–84 500) Ebola cases were averted in Sierra Leone as a direct result of additional treatment beds)	Lack of quality data on timing and role of different interventions
Ferrari et al. 2014 [47]	Meningitis (Nigeria)	Case management; strengthening of surveillance; mass vaccination campaigns	Healthcare provision; epidemiological investigation and/or surveillance	Reduction in confirmed meningitis cases	Positive (overall impact of vaccination campaigns ranged from 4 to 12%; vaccination reduced cases by as much as 50% when campaigns were conducted early in the epidemic)	Possible underestimation of campaign impact; provides an estimate of the vaccination campaign impact although rudimentary in its characterization of meningitis epidemiology
Yip et al. 2008 [48]	Severe acute respiratory syndrome (China)	Information dissemination to the public; quarantine; closure of most public facilities, schools and universities; site surveillance at airport	Epidemiological investigation and/or surveillance; Environmental and sanitary interventions	Daily numbers of confirmed SARS patients	Positive (drop in number of daily infections)	Only measured the aggregated effect of multiple intervention measures; only showed that number of cases started to decline at time of interventions; the study could not determine what measure was the most important factor leading to the reduction

*EVD* Ebola virus disease, *LF* Lassa fever, *SARS* Severe acute respiratory syndrome, *TB* Tuberculosis, *MDR-TB* Multi-drug resistant TB

^a^ The House index refers to the number of positive houses per total of inspected houses [23]

^b^ The Breteau index refers to the number of breeding sites per total of inspected houses [23]

### Quality of studies included

**Fig. 2:**
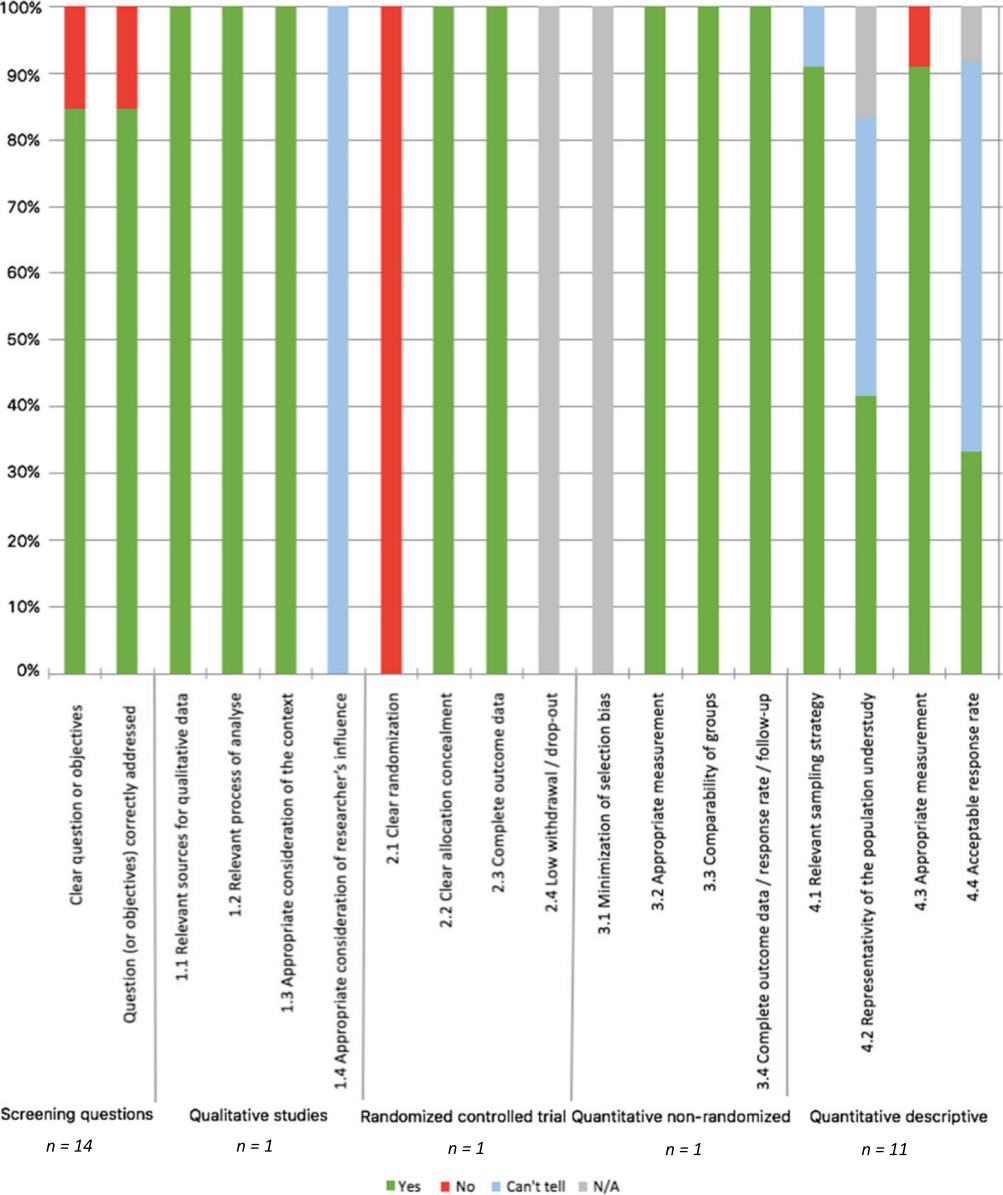
Quality assessment of studies according to the MMAT

Only 14 out of 31 articles were evaluated using the MMAT (see Fig. 2). Model-based (*n =* 7) and non-research case reports (*n =* 10) were excluded because the MMAT can only be used for experimental-type design intervention studies. The studies evaluated consisted of 11 descriptive studies, one randomized study, one case-control study, and one qualitative study. Overall, the quality of the studies evaluated was estimated as moderate, with a median score of 75%.

Among the 17 articles which could not be evaluated with the MMAT, 10 were classified as *non-research* [19–25, 27, 28, 49] as they did not provide a methods section, a description of the subjects, procedures or tools used, or a clear assessment of the outcomes. Given the lack of essential information to perform a quality assessment, these studies were considered to be of low quality.

### Types of containment measures

**Fig. 3:**
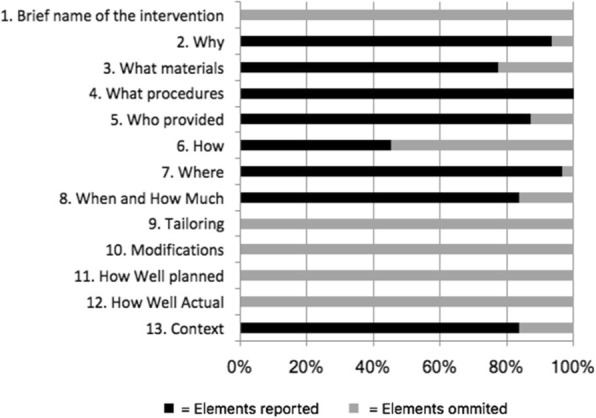
Percentage of studies reporting elements of description of the intervention according to the TIDieR tool

Figure 3 illustrates to what extent the interventions were described in each study. For the purpose of this paper, we distinguished between interventions and measures. Interventions were defined as a set of measures with a common objective, such as to achieve specific outcomes (i.e., the overall actions described in each study), and measures referred to the specific components of those interventions (i.e., each action developed in all studies). Measures were grouped into four categories: healthcare provision (*n =* 22, 71%), epidemiological investigation and/or surveillance (*n =* 19, 61%), environmental or sanitary measures (*n =* 19, 61%), and community-based measures (*n =* 9, 29%). The groupings were not mutually exclusive, therefore the percentage exceeded 100%.

*Healthcare provision measures* included: health infrastructure improvements, such as the construction of new clinics, treatment centres, or hospitals [20, 24, 28, 30, 34, 38, 39, 45, 48] (*n =* 9, 29%); improved case management [21, 27, 30, 33, 34, 36, 41] (*n =* 7, 23%); appointment of public health officers and reorientation of specialists [19, 22, 24, 28, 36, 48] (*n =* 6, 19%); training of doctors, community health workers, and other professionals [22, 28, 38, 39] (*n =* 4, 13%); provision of pre- or post-exposure prophylaxis [21, 30, 37] (*n =* 3, 10%); reactive vaccination campaigns [29, 41, 47] (*n =* 3, 10%); introduction of treatment beds [24, 46] (*n =* 2, 6%); use of new technology for diagnosis and treatment [24, 27] (*n =* 2, 6%); timely hospital admission and effective triage of patients [40] (*n =* 1, 3%), and safe transfer of identified cases [40] (*n =* 1, 3%). Psychosocial support was also provided in one study [25] (*n =* 1, 3%). Intervention materials included vaccines, medication, extra beds, and personal protective equipment [21, 24, 28–30, 37, 39, 41, 45–47] (*n =* 11, 35%).

*Epidemiological investigation and/or surveillance measures* consisted of: the setting up or improvement of surveillance systems [19, 24, 26, 27, 30, 33, 36, 37, 40, 49] (*n =* 10, 32%); active case-finding and contact tracing [25–28, 34, 37, 49] (*n =* 7, 23%); collection of serological samples and documentation of cases [22, 28, 37] (*n =* 3, 10%); entomological surveys and mapping [22–24] (*n =* 3, 10%); screening of all arrivals and departures in and out of the country by land, air, and sea [29, 49] (*n =* 2, 6%); operational studies for testing case definitions [22] (*n =* 1, 3%); and establishment of a phone-based alert management system [37] (*n =* 1, 3%). Intervention materials were real-time polymerase chain reaction (PCR), dashboards, and mobile phones [29, 44] (*n =* 2, 6%).

*Environmental and sanitary measures* focused mainly on the isolation or quarantine of symptomatic individuals or close contacts [25, 26, 29, 34–36, 38–40, 43, 48] (*n =* 11, 35%) and vector source reduction and chemical vector control measures in the case of vector-borne diseases [23, 24, 27, 31–33, 42, 44] (*n =* 8, 26%). Other types of measures included: decontamination of the environment [21, 23, 37, 38] (*n =* 4, 13%); safe burial practices [34, 36, 45] (*n =* 3, 10%); closing of public and entertainment facilities [39, 48] (*n =* 2, 6%); and water purification [24, 30] (*n =* 2, 6%). Campus quarantine was also used in one case [43] (*n =* 1, 3%). The intervention materials mostly consisted of larvicides, insecticides, insecticide treated bed nets, and material for water filtration and mosquito collection [23, 27, 31–33, 42, 50] (*n =* 7, 23%).

*Community-based measures* focused mainly on: involvement and training of community volunteers [33, 35, 36, 38, 49] (*n =* 5, 16%); awareness campaigns [20, 30, 31, 40, 41] (*n =* 5, 16%); and public education [31, 33, 41, 49] (*n =* 4, 13%). They also included community-based surveillance or case-finding [30, 35, 36] (*n =* 3, 10%) and social mobilization [22, 36] (*n =* 2, 6%). Materials used included pamphlets, posters, videos, social media platforms, and print and electronic media [24, 27, 35, 38, 49] (*n =* 5, 16%).

Lastly, most of the interventions were conducted only once, and duration ranged from one month and a half [25, 31, 43] to two years and four months [34]. No information was available on modifications made to the interventions during the study, on adherence of participants, or on intervention fidelity. The rationale for the majority of the studies was to focus on controlling the spread of disease and to mitigate further spread throughout the country. No conceptual theories were mentioned to justify the chosen interventions.

### Implementation process and transferability

**Fig. 4:**
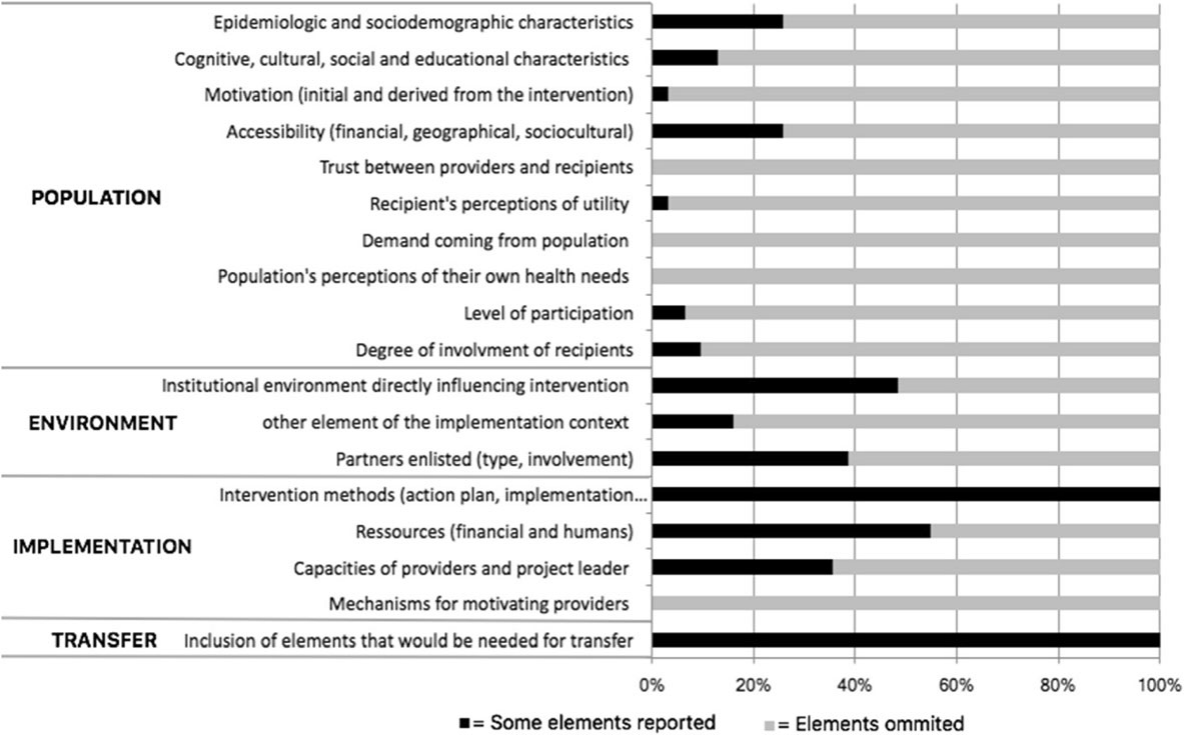
Percentage of studies with ASTAIRE elements

Using the ASTAIRE tool [18], as presented in the Methods section, we examined the availability of information on 23 elements related to the study’s population, environment, and implementation process, as well as elements needed for the intervention’s transfer (see Additional file 3 for all data extracted). Figure 4 illustrates the availability of those elements.

The recipient population was not well described in most studies, with only eight articles providing information on its epidemiologic and/or sociodemographic characteristics [21, 24, 27, 34, 37, 40, 41, 47], and four articles mentioned cognitive, social, and/or educational characteristics of the study population [17, 21, 35, 41]. The eight studies that evaluated the intervention’s financial, geographic, and/or sociocultural accessibility rated it as favourable [20, 24, 28, 30, 35, 37, 38, 41], although none of the studies described how these evaluations were made and are likely based on subjective opinion and information. The recipients’ perceptions of the intervention’s utility were mentioned in one study [35].

Institutional factors influencing the interventions, such as committed political will or decision-makers’ positive perceptions of the intervention, were rarely described. The types of partners involved with the intervention included international organizations (e.g. WHO, Médecins Sans Frontières, United Nation agencies), local and international non-governmental organizations, governmental institutions, and other stakeholders, such as local authorities or opinion leaders. The private sector (e.g. petrol, communications, and software companies) was also involved in one case [49]. Thirteen interventions were based on routine means and structures (e.g. local professionals and pre-existent infrastructures), while 11 relied on international assistance or the construction of new infrastructures, such as hospitals.

The different aspects related to the implementation process were minimally described in the majority of studies. The capacities of the providers and project leaders were only assessed in ten studies [19, 20, 24, 27, 33, 35, 38, 40, 48, 49], and among the nine articles that reported on financial resources, five mentioned a lack of funding [19, 20, 25, 28, 45]. In one study, the intervention was stopped after 14 weeks due to budget constraints [20].

### Evaluation of interventions

While most of the articles (24 out of 31) reported overall positive results, such as a reduction in disease burden or spread, seven studies reported neutral or negative results [23, 30, 32, 33, 35, 36, 44]. Outcomes used to evaluate the effectiveness of interventions varied largely among articles and included: number of cases [23, 24, 27, 29, 31, 33, 36, 37, 39, 41, 43–45, 47, 48]; case fatality rates [21, 23, 24, 29, 36, 38, 45]; entomological indices such as the House index and Breteau index [23, 31–33, 42]; delay in disease detection [36, 38] or time between illness onset and hospitalization [39, 40]; proportion of contacts among new cases [30]; development of resistant strains [30]; changes in the reproduction number [26]; and number of cases averted [46]. Four studies did not provide clear outcome measures [19, 22, 25, 26] while two studies controlled for measured confounders [41, 42], and seven used mathematical modeling approaches [26, 43–48]. Five studies assessed the effectiveness of specific measures rather than providing an average measure of effectiveness for the full intervention. One of those was a randomized study [42], one was a case-control study [41], and three were mathematical models [27, 38, 47].

### Challenges faced

The authors identified many challenges encountered in conducting containment measures. Eleven studies mentioned a lack of experience in the diagnosis, management, and treatment of the diseases in question among local doctors, mainly due to the non-endemicity of those diseases. This led to missing diagnoses and/or misdiagnosis of early cases, which delayed the time to outbreak identification and response [19, 22, 25, 27, 31, 33, 36–38, 40, 48]. Nine studies cited the absence of sufficient material resources and infrastructure as barriers to efficient containment of outbreaks [19, 20, 24, 28, 32, 33, 37, 38, 48]. Eight articles identified an important delay between the onset of the disease and the implementation of a response plan or access to treatment for infected individuals as an important challenge faced by the intervention [22, 24, 28, 34, 38, 39, 47, 49].

Urban settings were also mentioned as presenting particular challenges in nine instances [20, 23, 24, 30, 32, 35, 36, 39, 47], with urban epidemics considered more difficult to control than those occurring in rural areas. The reasons included: high population density [20, 23, 24, 32, 39, 47]; population mobility [30, 32, 35]; and rapid, unplanned urbanization [31, 32]. One article mentioned the lack of community involvement and absence of strong social networks as challenges related to infectious disease control in urban settings. Urban dwellers, as opposed to rural residents, were described as persons who are “individualistic, lack social support, and are money dependant and difficult to mobilise in their overcrowded neighbourhoods” [36]. Conversely, one article mentioned the higher education level of urban residents as well as the easiest availability of healthcare resources as urban factors for more efficient containment of diseases [20].

### Lessons learned and recommendations

Most articles provided recommendations for the effective containment of future diseases. Those included improving surveillance measures (*n =* 10, 32%), reducing the delay between disease onset and implementation of interventions (*n =* 9, 29%), involving the community in the intervention (*n =* 7, 23%), improving medical infrastructure and resources (*n =* 7, 23%), reinforcing the training of health professionals (*n =* 4, 13%), and developing and disseminating outbreak management guidelines (*n =* 4, 13%).

## Discussion

The aim of this study was to determine the scientific knowledge of the effectiveness of containment measures of emerging and re-emerging vector-borne and other infectious diseases of poverty in urban settings. We found that there is limited evidence of effectiveness, given the poor to moderate quality of the evaluation of the interventions which were focused on Ebola or dengue control, excluding several relevant infectious diseases. We have developed several recommendations for researchers and practitioners to improve the quality of evidence for containment measures.

There were a variety of containment measures used simultaneously for the control of emerging or re-emerging diseases of poverty in urban centres. In the majority of the studies, it was not possible to determine the effect of any single intervention due to their overlapping and concurrent implementation. The data supporting the evidence on the effectiveness of control measures were generally observational and rarely experimental with the designs of approximately one-third of the included studies being case reports of low methodological quality. Additionally, 65% of the studies did not specify their evaluation methodology, based their conclusions on limited data, and/or could not attribute the control of the outbreak to a specific intervention. While most studies provided recommendations for infectious disease control in urban centres, in most instances those were not supported by the appropriate data. They appeared to be subjectively-based rather than evidence-based recommendations, which highlights the need for a higher degree of scientific rigour to avoid the replication of unsuccessful strategies [51].

Multifactorial issues associated with conducting research in real-world settings, such as context-specific issues related to the implementation of the research project also inhibited the ability to evaluate the effectiveness of interventions. Real-world scenarios and settings present the challenge of adapting theoretical (and idealistic) strategies to practical (and sometimes far from ideal) scenarios [52], which usually interfere with the development, success, and therefore evaluation of intervention studies. Checklists such as the TIDieR and ASTAIRE can be used to document information regarding the description, implementation processes, and transferability of those interventions [16, 18]. For the studies included in this review, the quantity and the quality of information on the interventions’ implementation processes, as well as on intervention modifications and intervention fidelity, were insufficiently described. This lack of information is problematic considering the importance of gathering information on the implementation process of real-world interventions to provide a better account of complex phenomena [51]. As a result, the complexity of the context in which the interventions took place could not be assessed in most cases, thus drastically reducing the transferability of the interventions.

### Limitations of the study

As we only included articles published in English and French, relevant documents in other languages such Spanish, Portuguese, or native languages from the Asian region were not considered, which may have resulted in a differential exclusion of information from relevant settings. Additionally, no information was included on the measures taken in response to the Zika virus pandemic, due to the time at which our search strategy was performed. Although overall patterns and research gaps could be identified for the group of conditions studied, the implicit heterogeneity of the definition of ‘diseases of poverty’ presented a challenge when summarizing the results of our search. These issues were overcome using general checklist tools, but we acknowledge the usefulness of narrower definitions and specificity of topics.

### Implications for future research

This review has highlighted several knowledge gaps and priority needs for future research highlighted in Table 2. Firstly, future research should seek to work within real-world conditions rather than through controlled studies, as there is a need for research designs that take into account the complexity of the settings in which the interventions occur. This would allow the influencing factors of intervention implementation in complex systems (e.g., political support) to be considered. Similarly, longer follow-up periods and methodologically rigorous data collection would improve the quality of future studies. Mathematical models can be a valuable tool for informing control measures although the outputs of the models should be evaluated, such as applied to field settings.

**Table 2:** Knowledge gaps and priority needs for future research

• Future research designs need to account for the complexity of real-world settings • Longer follow-up times and more comprehensive data are needed to better understand causality and the effects of the interventions • Comprehensive and systematic descriptions of implementation processes are needed, as are descriptions of contextual elements related to transferability, following standardized reporting guidelines • More and better evidence is needed on control measures for neglected tropical diseases • The theoretical models underpinning interventions need to be strengthened • Evidence-based lessons and recommendations should be generated from research that is conducted objectively	

Secondly, there is a clear need for routine and systematic description of the implementation process, context, and related elements needed for transfer to future studies and scenarios [53]. Standardized reporting checklists such as TIDieR and ASTAIRE should be used in planning and reporting interventions in order to improve knowledge transfer between researchers and enable public health practitioners to reproduce the achieved results in future interventions. We also suggest that the use of these tools should be a requirement of scientific journals that publish research concerning public health interventions.

Thirdly, given that the largest body of evidence concerned control activities for Ebola virus and dengue fever, our study highlights the need to expand the body of evidence on the containment of neglected tropical diseases such as chikungunya, human African trypanosomiasis, and leishmaniosis.

Lastly, both researchers and public health practitioners would benefit from more theory-informed approaches for disease control [54, 55]. Studies based on mathematical models and implementation theories would help to define factors mediating the speed and effectiveness of containment measures and would improve the ability of public health practitioners to conduct informed interventions. Similarly, evidence-based lessons and recommendations are needed to enable the development of more useful policies and guidelines.

### Implications for public health policy and/or practice

Based on our findings, we highlighted implications for public health policy and practice which are summarized in Table 3. Ideally, public health practitioners should focus on proactive rather than reactive approaches. This would involve reinforcing the training of doctors and other health professionals on the diagnosis, management, and treatment of emerging and re-emerging diseases, increasing the resources available for disease containment, and improving medical infrastructure before the onset of any outbreak or epidemic. We recognize that such recommendations are not always feasible in low-resource settings, and this review identified other areas of practice that can be more easily addressed. Among them, the funding of post-intervention research and the inclusion of an evaluation period in the design of the intervention is needed. Similarly, future interventions should be planned on the basis of existing evidence and theory.

**Table 3:** Implications for public health policy and/or practice

• Focus on a proactive approach when time and resources allow: reinforcement of training, planning, and investments in materials • Fund post-intervention research • Include an adequate evaluation period in the planning of interventions • Rely on theory when planning interventions and making evidence-based recommendations • Promote comprehensive intervention description, especially regarding context, using validated checklists such as TIDieR and ASTAIRE	

## Conclusions

The results of this review demonstrate that there is an important lack of good quality evidence to guide infectious disease containment measures. The majority of interventions included this review were complex, which was further complicated by the setting or context where the intervention was implemented. There are actions that should be taken to improve the quality of the evidence and to account for the context through comprehensive and standard reporting. Allocating research efforts to evaluating the implementation processes of interventions is an important step in improving the control of emerging and re-emerging diseases.

## Additional files

Additional file 1: Multilingual abstracts in the five official working languages of the United Nations. (PDF 1047 kb)

Additional file 2: Complete search strategy. (DOCX 22 kb)

Additional file 3: Data extraction grid. (XLSX 104 kb)

## Abbreviations

ASTAIRE: Analysis of the transferability and support to adaptation of health promotion interventions
MMAT: Mixed Method Appraisal Tool
PRISMA: Preferred Reporting Items for Systematic Reviews and Meta-Analyses
TDR: the Special Programme for Research and Training in Tropical Diseases
TIDiER: Template for Intervention Description and Replication
VERDAS: VEctor boRne DiseAses Scoping reviews
WHO: World Health Organization
