# Supplementary material for: Containment measures for emerging and re-emerging vector-borne and other infectious diseases of poverty in urban settings: a scoping review
Source: Infect Dis Poverty. 2018 Sep 3;7:95. doi: 10.1186/s40249-018-0478-4 (PMC6120079; doi:10.1186/s40249-018-0478-4)
Supplement: Supplementary file 1 — Multilingual abstracts in the five official working languages of the United Nations. (PDF 1047 kb) [file 40249_2018_478_MOESM1_ESM.pdf]

Translation of the abstract into the five official working languages of the United Nations

تدابير الاحتواء لظهور وإعاده ظهور الامراض المنقولة بالنواقل وغيرها من الأمراض المعدية للفقر في البيئات الحضرية: مراجعة استعراضية

لورانس كامبيو ، وستيفاني ديغروتي ، وفاليري ريدي ، ومابيل كارابالي ، وكيت زينسر

نبذة مختصرة

معلومات أساسية: ويشكل ظهور وعودة ظهور الأمراض المنقولة بواسطة ناقلات الأمراض وغيرها من الأمراض المعدية للفقر خطرا على صحة السكان الذين يعيشون في البيئات الحضرية والمنخفضة الدخل. ومن الضروري التوصل إلى فهم مفصل للإجراءات التي تشمل فعالية احتواء تفشي المرض في الماضي من أجل تحسين التدخلات في المستقبل. وكان الهدف هو تحديد ما هو معروف عن فعالية تدابير الاحتواء لظهور وإعاده ظهور الأمراض المنقولة وغيرها من الأمراض المعدية للفقر في البيئات الحضرية وتحديد الثغرات في البحوث وأثارها على ممارسة الصحة العامة.

الجزء الرئيسي :أجرينا مراجعة استطلاعية وبحثنا بصورة منهجية عن مراجع تمت مراجعتها من قبل الأقران والمنشورات غير الرسمية التي نشرت بين عامي 2000 و 2016. . واستخدمت أدوات استخراج البيانات المختلفة لترميز البيانات واستخلاصها ، واستخلصت من جميع الدراسات بيانات عن عملية التنفيذ وقابلية النقل. أجري تقييم للجودة لكل دراسة مشمولة.

قمنا بفحص 205 من النصوص الكاملة للمقالات والتقارير لمجموع 31 من المقالات المدرجة في الاستعراض. وكانت جودة الدراسات منخفضه عموما إلى متوسطه. وكانت أكبر مجموعة من الأدلة تتعلق بأنشطة مكافحة فيروس إيبولا وحمى الضنك. واعتمدت غالبية التدخلات (87%) على أنواع متعددة من التدابير التي جمعت في أربع فئات: 1) توفير الرعاية الصحية ؛ 2) التحريات و/أو المراقبة الوبائية ؛ 3) التدخلات البيئية أو الصحية ؛ و 4) التدخلات المجتمعية. وكانت جودة غالبية الدراسات (90 %) ضعيفة أو معتدله ، ولم يقدم ثلث الدراسات وصفاً واضحاً للنتائج والإجراءات و/أو الأدوات المستخدمة للتدخل.

الاستنتاجات:وتسلط نتائجنا الضوء علي صعوبة إثبات السببية عند تقييم أثر تدابير الاحتواء. وهناك حاجة ملحة إلى إجراء دراسات تتجاوز مجرد الإبلاغ عن الفعالية وتأخذ في الاعتبار تعقد الأوضاع في العالم الحقيقي. ونوصي بتخصيص الجهود البحثية لتقييم عمليات تنفيذ التدخلات فضلاً عن وصفها الشامل والمنهجي باستخدام قوائم التحقق المصادق عليها.

Translated from English version into Arabic by Shefa'a Qudah, proofread by Rehab Rayan, through

TRANSLATORS
WITHOUT BORDERS

城市环境中新现和复现的媒传疾病及其他贫困所致传染病的控制措施：勘域综述

Laurence Campeau, Stéphanie Degroote, Valery Ridde, Mabel Carabali and Kate Zinszer

摘要

引言：媒传疾病和其他贫困所致传染病的出现和复现对生活在城市和低收入环境中的人群健康构成威胁。为改进未来干预措施，有必要详细了解包括暴发疫情控制有效措施在内的一系列干预行为。本文的目的在于明确城市环境中新现和复现的媒传疾病和其他贫困所致传染病已有控制措施的有效性，并确定研究差距及其对公共卫生实践的影响。

主要内容：我们对 2000-2016 年期间发表的同行评议和灰色文献进行勘域综述和系统检索。使用不同的数据提取工具进行数据编码和提取，并从所有研究中提取实施过程和可转移性的数据。对纳入的每项研究进行质量评估。

共筛选了 205 篇全文论文和报告，纳入 31 篇。研究的质量普遍处于低至中等。主体证据涉及埃博拉病毒病和登革热的控制措施。大多数干预行为（87%）依赖于多种措施，这些措施分为四类：（1）提供医疗保健、（2）流行病学调查和/或监测、（3）环境或卫生措施干预，和（4）以社区为基础的干预措施。大多数研究（90%）的质量很差或适中，1/3 的研究未对结果以及用于干预的程序和/或工具进行清晰描述。

结论：本研究结果强调在评估控制措施效果时难以确定因果关系。迫切需要不仅仅研究防治措施的有效性，还需考虑到现实环境的复杂性。我们建议将研究工作转向对干预措施的实施过程进行评估，并采用经过验证的清单对干预措施进行全面和系统的描述。

Translated from English version into Chinese by Peng Song, edited by Pin Yang

CHINESE CENTER FOR DISEASE CONTROL AND PREVENTION

NATIONAL INSTITUTE OF PARASITIC DISEASES

Mesures de confinement pour les maladies émergentes et ré-émergentes à transmission vectorielle et autres maladies infectieuses liées à la pauvreté en milieu urbain : un examen de la portée

Laurence Campeau, Stéphanie Degroote, Valery Ridde, Mabel Carabali et Kate Zinszer

Résumé

Contexte: L'émergence et la ré-émergence de maladies à transmission vectorielle et d'autres maladies infectieuses liées à la pauvreté menacent la santé des populations vivant dans les zones urbaines et à faible revenu. Une compréhension détaillée des actions, y compris l'efficacité de l'endiguement de l'épidémie est nécessaire pour améliorer les interventions futures. L'objectif était de déterminer ce que l'on sait de l'efficacité des mesures de confinement des maladies émergentes et ré-émergentes à transmission vectorielle et d'autres maladies infectieuses en milieu urbain, ainsi que d'identifier les lacunes et les implications de la recherche en santé publique.

Corps principal: Nous avons examiné la portée et systématiquement recherché tant la littérature évaluée par les pairs que la littérature grise publiée entre 2000 et 2016. Différents outils d'extraction de données ont été utilisés pour le codage et l'extraction des données. Par ailleurs, des données sur le processus de mise en œuvre et la transmissibilité ont été extraites de toutes les études. Une évaluation de la qualité a été effectuée pour chaque étude incluse.

Nous avons analysé 205 articles en texte intégral et des rapports pour un total de 31 articles inclus dans l'examen. La qualité des études était généralement faible à modérée. La majorité des interventions (87%) reposait sur plusieurs types de mesures, regroupées en quatre catégories : 1) prestation de soins de santé; 2) enquête épidémiologique et/ou surveillance; 3) les interventions environnementales ou sanitaires; et 4) les interventions communautaires. La qualité de la majorité des études (90%) était faible ou modérée, et un tiers des études ne fournissait pas de description claire des résultats et des procédures et/ou des outils utilisés pour l'intervention.

Conclusions: Nos résultats soulignent qu'il est difficile d'établir une causalité lors de l'évaluation de l'effet des mesures de confinement. Il est urgent de disposer d'études qui fassent plus que simplement discuter l'efficacité et prendre en compte la complexité des environnements réels. Nous recommandons l'attribution des efforts de recherche à l'évaluation des processus de mise en œuvre des interventions ainsi que leur description complète et systématique à l'aide de listes de contrôle validées.

Translated from English version into French by Iris Soliman, proofread by Suzanne Assenat, through

TRANSLATORS
WITHOUT BORDERS

Меры сдерживания впервые и повторно возникающих трансмиссивных и других инфекционных заболеваний в городских условиях: аналитический обзор

Лоуренс Кампо, Стефани Дегрут, Валери Ридде, Мейбл Карабали и Кейт Зинсцер

Аннотация

Предпосылки: Первое и повторное появление трансмиссионных и других инфекционных заболеваний бедности создает угрозу для здоровья населения, проживающего в городских и малообеспеченных районах. Для улучшения последующих мероприятий необходимо полностью понимать действия, в том числе эффективность мер сдерживания прошлых вспышек. Целью являлось определение того, что известно об эффективности мер сдерживания впервые и повторно возникающих трансмиссионных и других инфекционных заболеваниях бедности в городских условиях, а также выявление пробелов в исследованиях и последствия для медицинских услуг.

Основная часть: Для данного аналитического обзора был проведен поиск по рецензируемой и внеиздательской литературе, опубликованной между 2000 и 2016 гг. Для кодирования и извлечения данных применялись различные средства извлечения, тогда как по всем исследованиям также были получены данные о процессе внедрения и переносимости. Оценка качества проводилась по каждому включенному исследованию.

Были отобраны 205 полнотекстовых статей и отчетов, также в обзор была включена в общей сложности 31 статья. Общее качество исследований варьировалось от низкого до среднего. Наибольшая доказательственная база касалась мер сдеживания вируса Эбола и лихорадки денге. Большинство (87%) мероприятий основывались на нескольких типах мер, которые были сгруппированы в четыре категории: 1) предоставление медицинской помощи; 2) эпидемиологическое исследование и (или) наблюдение; 3) экологические или санитарные меры; и 4) мероприятия на уровне общин. Качество большинства исследований (90%) варьировалось от низкого до среднего, а одна треть исследований не давала четкого описания результатов и процедур и (или) инструментов, используемых для мероприятий.

Выводы: Полученные результаты подчеркивают сложности, возникающие при выявлении причин в процессе оценки влияния мер сдерживания. Крайне необходимы исследования, которые выходят за рамки самой лишь отчетности об эффективности и учитывают сложность реальных ситуаций. Рекомендовано распределять комплекс исследований для оценки процессов реализации мероприятий, а также их всестороннего и систематического описания с использованием проверенных контрольных списков.

Translated from English version into Russian by Halyna Maksymiv, proofread by Liudmila Tomanek, through

TRANSLATORS
WITHOUT BORDERS

Las medidas de contención para el surgimiento y resurgimiento de enfermedades infecciosas transmitidas por vectores y otras enfermedades infecciosas que afectan a aquellos en situación de pobreza: una evaluación de campo.

Laurence Campeau, Stéphanie Degroote, Valery Ridde, Mabel Carabali and Kate Zinszer

Resumen

Antecedentes: El surgimiento y resurgimiento de enfermedades infecciosas transmitidas por vectores, entre otras enfermedades infecciosas que afectan a aquellos en situación de pobreza, son una amenaza para las poblaciones sanas que viven en entornos urbanos y de ingresos bajos. Para comprender mejor las acciones entre las cuales se encuentra la efectividad en la contención de brotes infecciosos anteriores es necesario para poder mejorar intervenciones futuras. El objetivo fue determinar qué se sabe sobre la efectividad de las medidas de contención para el surgimiento y resurgimiento de enfermedades infecciosas transmitidas por vectores y otras enfermedades infecciosas que afectan a aquellos en situación de pobreza en contextos urbanos, e identificar la brechas dentro de la investigación además de las consecuencias que pudieran tener para la práctica de la salud pública.

Cuerpo principal: Realizamos una revisión de campo y una búsqueda sistemática de la literatura, tanto revisada por pares como literatura gris, publicada entre el 2000 y el 2016. Se utilizaron diferentes herramientas de extracción de datos para la codificación y extracción de los datos, y se extrajo información acerca de el proceso de implementación y la transferabilidad de todos los estudios. Se realizó una evaluación de la calidad de cada estudio incluido.

Se evaluaron 205 artículos completos y reportes, dando un total de 31 artículos incluidos en la revisión. La calidad de los estudios fue, en términos generales, de baja a moderada. El mayor conjunto de pruebas estaba relacionado con las actividades de control del virus del Ébola y la fiebre de dengue. La mayoría de la intervenciones (87%) se basaban en diversos tipos de medidas, que se agrupan en cuatro categorías: 1) asistencia sanitaria; 2)investigación o control epidemiológico; 3) intervenciones ambientales o sanitarias; y 4) intervenciones basadas en la comunidad. La calidad de la mayoría de los estudios (90%) era pobre o moderada, y un tercio de los estudios no daba una descripción clara de los resultados ni de los procedimientos o herramientas utilizadas en la intervención.

Conclusión: Los resultados destacan la dificultad para establecer causalidad al evaluar el efecto de las medidas de contención. Se necesitan urgentemente estudios que vayan más allá de simplemente reportar la efectividad y que tengan en cuenta la complejidad de los escenarios reales. Recomendamos que se asignen actividades de investigación destinadas a la evaluación de los procesos de implementación de intervenciones así como también descripciones completas y sistemáticas con la utilización de listas de control validadas.

Translated from English version into Spanish by E Passadore, proofread by Angeles Possetti, through

TRANSLATORS
WITHOUT BORDERS
