# Supplementary material for: Containment measures for emerging and re-emerging vector-borne and other infectious diseases of poverty in urban settings: a scoping review
Source: Infect Dis Poverty. 2018 Sep 3;7:95. doi: 10.1186/s40249-018-0478-4 (PMC6120079; doi:10.1186/s40249-018-0478-4)
Supplement: Supplementary file 2 — Complete search strategy. (DOCX 22 kb) [file 40249_2018_478_MOESM2_ESM.docx]

**Search strategy form**

3.1 Search for Medline

Vector-borne disease$.ti,ab,kw. or mosquito-borne disease$.ti,ab,kw. or (infectious ADJ2 disease$.ti,ab,kw.) or emerging infectious disease$.ti,ab,kw. or re-emerging infectious disease$.ti,ab,kw. or communicable disease$.ti,ab,kw. or emerging communicable disease$.ti,ab,kw. or reemerging communicable disease$.ti,ab,kw. or neglected tropical disease$.ti,ab,kw. or disease vector$.ti,ab,kw. or insect vector$.ti,ab,kw. or arthropod vector$.ti,ab,kw. or aracnid vector$.ti,ab,kw. or tropical disease$.ti,ab,kw. or borreliosis.ti,ab,kw. or tularaemia.ti,ab,kw. or american trypanosomiasis.ti,ab,kw. or malaria.ti,ab,kw. or dengue.ti,ab,kw. or rift valley fever.ti,ab,kw. or yellow fever.ti,ab,kw. or zika.ti,ab,kw. or japanese encephalitis.ti,ab,kw. or lymphatic filariasis.ti,ab,kw. or West Nile fever.ti,ab,kw. or Schistosomiasis.ti,ab,kw. or Sandfly fever.ti,ab,kw. or phelebotomus fever.ti,ab,kw. or bilharziasis.ti,ab,kw. or filariasis.ti,ab,kw. or chikungunya.ti,ab,kw. or leishmaniasis.ti,ab,kw. or Crimean-Congo haemorrhagic fever.ti,ab,kw. or Lyme disease.ti,ab,kw. or Relapsing fever.ti,ab,kw. or borreliosis.ti,ab,kw. or Rickettsial diseases.ti,ab,kw. or spotted fever.ti,ab,kw. or Q fever.ti,ab,kw. or Tick-borne encephalitis.ti,ab,kw. or Tularaemia.ti,ab,kw. or Chagas disease.ti,ab,kw. or American trypanosomiasis.ti,ab,kw. or Sleeping sickness.ti,ab,kw. or African trypanosomiasis.ti,ab,kw. or Plague.ti,ab,kw. or Rickettsiosis.ti,ab,kw. or Onchocerciasis.ti,ab,kw. or river blindness.ti,ab,kw. or Rabies.ti,ab,kw. or trachoma.ti,ab,kw. or buruli ulcer.ti,ab,kw. or endemic treponematoses.ti,ab,kw. or food-borne trematodiases.ti,ab,kw. or echinococcosis.ti,ab,kw. or leprosy.ti,ab,kw. or cysticercosis.ti,ab,kw. or tuberculosis.ti,ab,kw. or Filovirus diseases.ti,ab,kw. or ebola virus.ti,ab,kw. or marburg.ti,ab,kw. or lassa fever.ti,ab,kw. or Middle East Respiratory Syndrome.ti,ab,kw. or mers.ti,ab,kw. or Severe acute respiratory syndrome.ti,ab,kw. or sars.ti,ab,kw. or nipah.ti,ab,kw. or avian influenza.ti,ab,kw. or Coccidioides immitis.ti,ab,kw. or Coccidioides posadasii.ti,ab,kw. Or cholera.ti,ab,kw. Or (Meningococcal meningitis or Legionella pneumonia or Hepatitis B or hepatitis C or Multidrug-resistant TB or Viral encephalitis or Hendra virus or Hantavirus haemorrhagic fever or cardiopulmonary syndrome or Escherichia coli or Gastroenteritis due to norovirus or Legionella pneumonia).ti,ab,kw. or Communicable Diseases, Emerging.sh. or Disease Vectors.sh. or Disease transmission, Infectious.sh. or Neglected Diseases.sh. or Communicable Diseases.sh. or Insect Vectors.sh. or Arthropod vectors.sh. or Sandfly fever Naples virus.sh. or Phlebovirus.sh. or Phlebotomus fever.sh. or Schistosomiasis.sh. or Filariasis.sh. or Chikungunya virus.sh. or Chikungunya fever.sh. or Leishmaniasis.sh. or Leishmaniasis, Visceral.sh. or Hemorrhagic Fever Virus.sh. or Crimean-Congo.sh. or Hemorrhagic Fever, Crimean.sh. or Lyme disease.sh. or Relapsing fever.sh. or Borrelia Infections.sh. or Rickettsia.sh. or Rickettsia Infections.sh. or Q fever.sh. or Coxiella burneti.sh. or Encephalitis Viruses.sh. or Tick-Borne.sh. or Encephalitis, tick-borne.sh. or Tularaemia.sh. or Chagas disease.sh. or Trypanosomiasis, African.sh. or Malaria.sh. or Dengue.sh. or Rift valley fever.sh. or Rift Valley fever virus.sh. or Yellow fever.sh. or Zika virus infection.sh. or Zika virus.sh. or Encephalitis, Japanese.sh. or Elephantiasis, Filarial.sh. or West nile fever.sh. or Schistosomiasis.sh. or Plague.sh. or Yersinia pestis.sh. or Onchocerciasis.sh. or Onchocerciasis, ocular.sh. or Rabies.sh. or Trachoma.sh. or buruli ulcer.sh. or echinococcosis.sh. or leprosy.sh. or cysticercosis.sh. or tuberculosis.sh. or Ebolavirus.sh. or Hemorrhagic Fever, Ebola.sh. or marburg virus disease.sh. or Marburgvirus.sh. or Lassa Fever.sh. or Coronavirus Infections.sh. or Middle East Respiratory Syndrome Coronavirus.sh. or Severe acute respiratory syndrome.sh. or Nipah Virus.sh. or Coccidioides.sh. or Cholera.sh. (570479 results)

AND

Urban$.ti,ab,kw. or city.ti,ab,kw. Or cities.ti,ab,kw. or metropolitan area$.ti,ab,kw. or metropolitan.ti,ab,kw. or non?rural.ti,ab,kw. or neighbo?rhood$.ti,ab,kw. or downtown.ti,ab,kw. or midtown.ti,ab,kw. or uptown.ti,ab,kw. or building$.ti,ab,kw. or district$.ti,ab,kw. or slum$.ti,ab,kw. or barrio$.ti,ab,kw. or township$.ti,ab,kw. or city center.ti,ab,kw. or municipal$.ti,ab,kw. or civic.ti,ab,kw. or densely populated.ti,ab,kw. or suburban.ti,ab,kw. or town$.ti,ab,kw. or informal settlement$.ti,ab,kw. Or Urban Population.sh. or Urban health.sh. or Cities.sh. (385903 results)

AND

epidemic$.ti,ab,kw. or pandemic$.ti,ab,kw. or outbreak$.ti,ab,kw. or emergenc$.ti,ab,kw. or surge.ti,ab,kw. or re-occurrence.ti,ab,kw. or emergency relief.ti,ab,kw. or detection.ti,ab,kw. Or re?emergence.ti,ab,kw. or Epidemics.sh. or Pandemics.sh. or disease outbreaks.sh. or Emergencies.sh. (1007844 results)

AND

Containment measure$.ti,ab,kw. or control measure$.ti,ab,kw. or infection control.ti,ab,kw. or (control$ ADJ4 disease$).ti,ab,kw. or control$ strateg$.ti,ab,kw. or epidemic control.ti,ab,kw. or intervention measure$.ti,ab,kw. or control strateg$.ti,ab,kw. or control$.ti,ab,kw. or transmission containment.ti,ab,kw. or transmission control.ti,ab,kw. or (stop$ ADJ3 epidemic$).ti,ab,kw. or disease-control measure$.ti,ab,kw. or emergency response$.ti,ab,kw. or response intervention$.ti,ab,kw. or eliminat$.ti,ab,kw. or eradicat$.ti,ab,kw. or Emergency Preparedness.ti,ab,kw. or operational response$.ti,ab,kw. or mosquito$ control.ti,ab,kw. or interruption.ti,ab,kw. or Infection Control.sh. or Communicable Disease Control.sh. (2998219 results)

AND

(Evaluat$ or rapidity or efficacity or effectiveness or outcome$ or impact$ or effect$).ti,ab,kw. or Program Evaluation.sh. or Comparative Effectiveness Research.sh. or Evaluation Studies.sh. or "Outcome and Process Assessment (Health Care)".sh. or "Outcome Assessment (Health Care)".sh. (7272615 results)

Résultats combinés: 1430

3.2. Search for Embase

Vector-borne disease$.ti,ab,kw. or mosquito-borne disease$.ti,ab,kw. or (infectious ADJ2 disease$).ti,ab,kw. or emerging infectious disease$.ti,ab,kw. or re-emerging infectious disease$.ti,ab,kw. or communicable disease$.ti,ab,kw. or emerging communicable disease$.ti,ab,kw. or reemerging communicable disease$.ti,ab,kw. or neglected tropical disease$.ti,ab,kw. or disease vector$.ti,ab,kw. or insect vector$.ti,ab,kw. or arthropod vector$.ti,ab,kw. or aracnid vector$.ti,ab,kw. or tropical disease$.ti,ab,kw. or borreliosis.ti,ab,kw. or tularaemia.ti,ab,kw. or american trypanosomiasis.ti,ab,kw. or malaria.ti,ab,kw. or dengue.ti,ab,kw. or rift valley fever.ti,ab,kw. or yellow fever.ti,ab,kw. or zika.ti,ab,kw. or japanese encephalitis.ti,ab,kw. or lymphatic filariasis.ti,ab,kw. or West Nile fever.ti,ab,kw. or Schistosomiasis.ti,ab,kw. or Sandfly fever.ti,ab,kw. or phelebotomus fever.ti,ab,kw. or bilharziasis.ti,ab,kw. or filariasis.ti,ab,kw. or chikungunya.ti,ab,kw. or leishmaniasis.ti,ab,kw. or Crimean-Congo haemorrhagic fever.ti,ab,kw. or Lyme disease.ti,ab,kw. or Relapsing fever.ti,ab,kw. or borreliosis.ti,ab,kw. or Rickettsial diseases.ti,ab,kw. or spotted fever.ti,ab,kw. or Q fever.ti,ab,kw. or Tick-borne encephalitis.ti,ab,kw. or Tularaemia.ti,ab,kw. or Chagas disease.ti,ab,kw. or American trypanosomiasis.ti,ab,kw. or Sleeping sickness.ti,ab,kw. or African trypanosomiasis.ti,ab,kw. or Plague.ti,ab,kw. or Rickettsiosis.ti,ab,kw. or Onchocerciasis.ti,ab,kw. or river blindness.ti,ab,kw. or Rabies.ti,ab,kw. or trachoma.ti,ab,kw. or buruli ulcer.ti,ab,kw. or endemic treponematoses.ti,ab,kw. or food-borne trematodiases.ti,ab,kw. or echinococcosis.ti,ab,kw. or leprosy.ti,ab,kw. or cysticercosis.ti,ab,kw. or tuberculosis.ti,ab,kw. or Filovirus diseases.ti,ab,kw. or ebola virus.ti,ab,kw. or marburg.ti,ab,kw. or lassa fever.ti,ab,kw. or Middle East Respiratory Syndrome.ti,ab,kw. or mers.ti,ab,kw. or Severe acute respiratory syndrome.ti,ab,kw. or sars.ti,ab,kw. or nipah.ti,ab,kw. or avian influenza.ti,ab,kw. or Coccidioides immitis.ti,ab,kw. or Coccidioides posadasii.ti,ab,kw. Or cholera.ti,ab,kw. Or (Meningococcal meningitis or Legionella pneumonia or Hepatitis B or hepatitis C or Multidrug-resistant TB or Viral encephalitis or Hendra virus or Hantavirus haemorrhagic fever or cardiopulmonary syndrome or Escherichia coli or Gastroenteritis due to norovirus or Legionella pneumonia).ti,ab,kw. or Communicable Diseases.sh. or Vector Control.sh. or Parasite Vector.sh. or Tropical disease.sh. or Schistosomiasis.sh. or Onchocerciasis.sh. or Lymphatic filariasis.sh. or Dengue.sh. or Helminthiasis.sh. or Parasite Vector.sh. or borrelia infection.sh. or Tularaemia.sh. or Chagas disease.sh. or Malaria .sh. or Rift Valley Fever.sh. or Arbovirus.sh. or Yellow fever.sh. or Flavivirus.sh. or Japanese encephalitis.sh. or West Nile fever.sh. or Sandfly fever.sh. or Filariasis.sh. or Chikungunya.sh. or Chikungunya virus.sh. or leishmaniasis.sh. or Crimean-Congo haemorrhagic fever.sh. or Lyme disease.sh. or Rickettsiosis.sh. or Q fever.sh. or Tick-borne encephalitis.sh. or African trypanosomiasis.sh. or Plague.sh. or Rabies.sh. or Rabies Virus.sh. or Trachoma.sh. or buruli ulcer.sh. or Treponematosis.sh. or echinococcosis.sh. or Leprosy.sh. or cysticercosis.sh. or tuberculosis.sh. or Ebola virus.sh. or Filovirus infection.sh. or Filoviridae.sh. or Virus hemorrhagic fever.sh. or Marburg hemorrhagic fever.sh. or Marburg virus.sh. or lassa fever.sh. or Middle East Respiratory Syndrome coronavirus.sh. or Coronavirus infection.sh. or Coronavirus.sh. or Severe acute respiratory syndrome.sh. or Nipah virus.sh. or Nipah virus infection.sh. or Coccidioides immitis.sh. or Coccidioides.sh. or Cholera.sh. (695799 results)

AND

Urban$.ti,ab,kw. or city.ti,ab,kw. Or cities.ti,ab,kw. or metropolitan area$.ti,ab,kw. or metropolitan.ti,ab,kw. or non?rural.ti,ab,kw. or neighbo?rhood$.ti,ab,kw. or downtown.ti,ab,kw. or midtown.ti,ab,kw. or uptown.ti,ab,kw. or building$.ti,ab,kw. or district$.ti,ab,kw. or slum$.ti,ab,kw. or barrio$.ti,ab,kw. or township$.ti,ab,kw. or city center.ti,ab,kw. or municipal$.ti,ab,kw. or civic.ti,ab,kw. or densely populated.ti,ab,kw. or suburban.ti,ab,kw. or town$.ti,ab,kw. or informal settlement$.ti,ab,kw. Or Urban area.sh. or Urban population.sh. or City.sh. (537658 results)

AND

epidemic$.ti,ab,kw. or pandemic$.ti,ab,kw. or outbreak$.ti,ab,kw. or emergenc$.ti,ab,kw. or surge.ti,ab,kw. or re-occurrence.ti,ab,kw. or emergency relief.ti,ab,kw. or detection.ti,ab,kw. Or re?emergence.ti,ab,kw. or Epidemic.sh. or Pandemic.sh. or Emergency.sh. (1401488 results)

AND

Containment measure$.ti,ab,kw. or control measure$.ti,ab,kw. or infection control.ti,ab,kw. or (control$ ADJ4 disease$).ti,ab,kw. or control$ strateg$.ti,ab,kw. or epidemic control.ti,ab,kw. or intervention measure$.ti,ab,kw. or control strateg$.ti,ab,kw. or control$.ti,ab,kw. or transmission containment.ti,ab,kw. or transmission control.ti,ab,kw. or (stop$ ADJ3 epidemic$).ti,ab,kw. or disease-control measure$.ti,ab,kw. or emergency response$.ti,ab,kw. or response intervention$.ti,ab,kw. or eliminat$.ti,ab,kw. or eradicat$.ti,ab,kw. or Emergency Preparedness.ti,ab,kw. or operational response$.ti,ab,kw. or mosquito$ control.ti,ab,kw. or interruption.ti,ab,kw. or Infection control.sh. or Disease control.sh. or Malaria control.sh. or Vector Control.sh. (4212101 results)

AND

(Evaluat$ or rapidity or efficacity or effectiveness or outcome$ or impact$ or effect$).ti,ab,kw. or Program effectiveness.sh. or Outcome assessment.sh. or Comparative Effectiveness.sh. or Evaluation Study.sh. or Evaluation Research.sh. or Outcome assessment.sh. or Program evaluation.sh. or Program efficacy.sh. (10229706 results)

Résultats combinés: 1897

3.3 Search for Global Health

Vector-borne disease$.ti,ab. or mosquito-borne disease$.ti,ab. or infectious disease$.ti,ab. or emerging infectious disease$.ti,ab. or re-emerging infectious disease$.ti,ab. or communicable disease$.ti,ab. or emerging communicable disease$.ti,ab. or reemerging communicable disease$.ti,ab. or neglected tropical disease$.ti,ab. or disease vector$.ti,ab. or insect vector$.ti,ab. or arthropod vector$.ti,ab. or aracnid vector$.ti,ab. or tropical disease$.ti,ab. or mosquito control.ti,ab. or borreliosis.ti,ab. or tularaemia.ti,ab. or american trypanosomiasis.ti,ab. or malaria.ti,ab. or dengue.ti,ab. or rift valley fever.ti,ab. or yellow fever.ti,ab. or zika.ti,ab. or japanese encephalitis.ti,ab. or lymphatic filariasis.ti,ab. or West Nile fever.ti,ab. or Schistosomiasis.ti,ab. or Sandfly fever.ti,ab. or phelebotomus fever.ti,ab. or bilharziasis.ti,ab. or filariasis.ti,ab. or chikungunya.ti,ab. or leishmaniasis.ti,ab. or Crimean-Congo haemorrhagic fever.ti,ab. or Lyme disease.ti,ab. or Relapsing fever.ti,ab. or borreliosis.ti,ab. or Rickettsial diseases.ti,ab. or spotted fever.ti,ab. or Q fever.ti,ab. or Tick-borne encephalitis.ti,ab. or Tularaemia.ti,ab. or Chagas disease.ti,ab. or American trypanosomiasis.ti,ab. or Sleeping sickness.ti,ab. or African trypanosomiasis.ti,ab. or Plague.ti,ab. or Rickettsiosis.ti,ab. or Onchocerciasis.ti,ab. or river blindness.ti,ab. or Rabies.ti,ab. or trachoma.ti,ab. or buruli ulcer.ti,ab. or endemic treponematoses.ti,ab. or food-borne trematodiases.ti,ab. or echinococcosis.ti,ab. or leprosy.ti,ab. or cysticercosis.ti,ab. or tuberculosis.ti,ab. or Filovirus diseases.ti,ab. or ebola virus.ti,ab. or marburg.ti,ab. or lassa fever.ti,ab. or Middle East Respiratory Syndrome.ti,ab. or mers.ti,ab. or Severe acute respiratory syndrome.ti,ab. or sars.ti,ab. or nipah.ti,ab. or avian influenza.ti,ab. or Coccidioides immitis.ti,ab. or Coccidioides posadasii.ti,ab. or (Meningococcal meningitis or Legionella pneumonia or Hepatitis B or hepatitis C or Multidrug-resistant TB or Viral encephalitis or Hendra virus or Hantavirus haemorrhagic fever or cardiopulmonary syndrome or Escherichia coli or Gastroenteritis due to norovirus or Legionella pneumonia).ti,ab. or Vector-borne diseases.sh. or Disease Vectors.sh. or Mosquito-borne diseases.sh. or Infectious Diseases.sh. or Emerging Infectious Diseases.sh. or Sandfly fever.sh. or Schistosomiasis.sh. or Filariasis.sh. or Chikungunya virus.sh. or Leishmaniasis.sh. or Crimean-Congo haemorrhagic fever virus.sh. or Arboviruses.sh. or Lyme disease.sh. or Relapsing fever.sh. or Borrelia burgdorferi.sh. or Rickettsial diseases.sh. or Rickettsia.sh. or Spotted fever.sh. or Tickborne diseases.sh. or Q fever.sh. or Tick-Borne encephalitis virus.sh. or Tularaemia.sh. or Chagas' disease.sh. or African Trypanosomiasis.sh. or Malaria.sh. or Dengue.sh. or Rift Valley Fever.sh. or Yellow Fever.sh. or Zika virus.sh. or Japanese encephalitis.sh. or Japanese encephalitis virus.sh. or Flavivirus.sh. or Zika Virus.sh. or West Nile fever.sh. or Schistosomiasis.sh. or Plague.sh. or Onchocerciasis.sh. or Rabis.sh. or Rabies virus.sh. or trachoma.sh. or buruli ulcer.sh. or treponematosis.sh. or echinococcosis.sh. or leprosy.sh. or cysticercosis.sh. or tuberculosis.sh. or Marburgvirus.sh. or Ebolavirus.sh. or Lassa fever.sh. or severe acute respiratory syndrome.sh. or Nipah virus.sh. or coccidioides immitis.sh. or Coccidioides.sh. or Cholera.sh. (552419 results)

AND

Urban$.ti,ab. or city.ti,ab. Or cities.ti,ab. or metropolitan area$.ti,ab. or metropolitan.ti,ab. or non?rural.ti,ab. or neighbo?rhood$.ti,ab. or downtown.ti,ab. or midtown.ti,ab. or uptown.ti,ab. or building$.ti,ab. or district$.ti,ab. or slum$.ti,ab. or barrio$.ti,ab. or township$.ti,ab. or city center.ti,ab. or municipal$.ti,ab. or civic.ti,ab. or densely populated.ti,ab. or suburban.ti,ab. or town$.ti,ab. or informal settlement$.ti,ab. Or Urban areas.sh. or Urban environment.sh. or Urban sites.sh. or Urban society.sh. or Urban population.sh. or Towns.sh. (238769 results)

AND

epidemic$.ti,ab. or pandemic$.ti,ab. or outbreak$.ti,ab. or emergenc$.ti,ab. or surge.ti,ab. or re-occurrence.ti,ab. or emergency relief.ti,ab. or detection.ti,ab. Or re?emergence.ti,ab. or Epidemics.sh. or Outbreaks.sh. or Emergencies.sh. or Emergency relief.sh. (301167 results)

AND

Containment measure$.ti,ab. or control measure$.ti,ab. or infection control.ti,ab. or (control$ ADJ4 disease$).ti,ab. or control$ strateg$.ti,ab. or epidemic control.ti,ab. or intervention measure$.ti,ab. or control strateg$.ti,ab. or control$.ti,ab. or transmission containment.ti,ab. or transmission control.ti,ab. or (stop$ ADJ3 epidemic$).ti,ab. or disease-control measure$.ti,ab. or emergency response$.ti,ab. or response intervention$.ti,ab. or eliminat$.ti,ab. or eradicat$.ti,ab. or Emergency Preparedness.ti,ab. or operational response$.ti,ab. or mosquito$ control.ti,ab. or interruption.ti,ab. or Disease Control.sh. or Control programmes.sh. or Vector control.sh. or Control Method.sh. or Intervention.sh. (687596 results)

AND

(Evaluat$ or rapidity or efficacity or effectiveness or outcome$ or impact$ or effect$).ti,ab. or Evaluation.sh. or Program evaluation.sh. or Program Effectiveness.sh. or efficacy.sh. or Impact.sh. or Effects.sh. (1397629 results)

Résultats combinés: 1919

3.4 Search for Web of Science

(TS=("Vector-borne disease*" OR "mosquito-borne disease*" OR "infectious NEAR/1 disease*" OR "emerging infectious disease*" OR "re-emerging infectious disease*" OR "communicable disease*" OR "emerging communicable disease*" OR "reemerging communicable disease*" OR "neglected tropical disease*" OR "disease Vector*" OR "Insect vector*" OR "arthropod vector*" OR "aracnid vector*" OR "tropical disease*" OR "borreliosis" OR "tularaemia" OR " american trypanosomiasis" OR "malaria" OR "dengue" OR " rift valley fever" OR "yellow fever" OR "zika" OR "japanese encephalitis" OR "lymphatic filariasis " OR "communicable disease*" OR "emerging communicable disease*" OR "reemerging communicable disease*" OR "neglected tropical disease*" OR "disease Vector*" OR "West Nile fever" OR "Schistosomiasis" OR "Sandfly fever" OR "phelebotomus fever" OR "bilharziasis" OR "filariasis" OR "chikungunya" OR "leishmaniasis" OR "Crimean-Congo haemorrhagic fever" OR "Lyme disease" OR "Relapsing fever" OR "borreliosis" OR "Rickettsial diseases" OR "spotted fever" OR "Q fever" OR "Tick-borne encephalitis" OR "Tularaemia" OR "Chagas disease" OR "American trypanosomiasis" OR "Sleeping sickness" OR "African trypanosomiasis" OR "Plague" OR "rickettsiosis" OR "Onchocerciasis" OR "river blindness" OR "Rabies" OR "trachoma" OR "buruli ulcer" OR "endemic treponematoses" OR "food-borne trematodiases" OR "echinococcosis" OR "leprosy" OR "cysticercosis" OR "tuberculosis" OR "filovirus diseases" OR "ebola virus" OR "marburg" OR "lassa fever" OR "Middle East Respiratory Syndrome" OR "mers" OR "Severe acute respiratory syndrome" OR "sars" OR "nipah" OR "avian influenza" OR "Coccidioides immitis" OR "Coccidioides posadasii" OR "cholera")) AND LANGUAGE: ("English" OR "French") (374,909 results)

AND

(TS= (Urban$ OR "city" OR "cities" OR "metropolitan area$" OR "metropolitan" OR "non?rural" OR "neighbo?rhood$" OR "downtown" OR "midtown" OR "uptown" OR "building$" OR "district$" OR "slum$" OR "barrio$" OR "township$" OR "city center" OR "municipal$" OR "civic" OR "densely populated" OR "suburban" OR "town$" OR "informal settlement$")) AND LANGUAGE: (English OR French) (999,334 results)

AND

(TS= ("epidemic$" OR "pandemic$" OR "outbreak$" OR "emergenc$" OR "surge" OR "re-occurrence" OR "emergency relief" OR "detection")) AND LANGUAGE: (English OR French) (1,357,112 results)

AND

(TS= ("Containment measure*" OR "control measure*" OR "infection control" OR "control* NEAR/4 disease*" OR "control* strateg*" OR "epidemic* control" OR "intervention measure*" OR "control strateg*" OR "control*" OR "transmission containment" OR "transmission control" OR "stop* NEAR/3 epidemic*" OR "disease-control measure*" OR "emergency response*" OR "response intervention*" OR "Disease Control" OR "eliminat*" OR "eradicat*" OR "Emergency Preparedness" OR "operational response*" or "mosquito* control")) AND LANGUAGE: (English OR French) (4,909,096 results)

AND

(TS= ("Evaluat$" or "rapidity" or "efficacy" or "effectiveness" or "outcome*" or "outcome NEAR/3 assessment" or "impact" or "effects" or "Program Evaluation")) AND LANGUAGE: (English OR French) (6,917,599 results)

Résultats combinés: 528

3.5 Search for Cochrane Database of Systematic Reviews

Vector-borne disease$.ti,ab,kw. or mosquito-borne disease$.ti,ab,kw. or (infectious ADJ2 disease$.ti,ab,kw.) or emerging infectious disease$.ti,ab,kw. or re-emerging infectious disease$.ti,ab,kw. or communicable disease$.ti,ab,kw. or emerging communicable disease$.ti,ab,kw. or reemerging communicable disease$.ti,ab,kw. or neglected tropical disease$.ti,ab,kw. or disease vector$.ti,ab,kw. or insect vector$.ti,ab,kw. or arthropod vector$.ti,ab,kw. or aracnid vector$.ti,ab,kw. or tropical disease$.ti,ab,kw. or borreliosis.ti,ab,kw. or tularaemia.ti,ab,kw. or american trypanosomiasis.ti,ab,kw. or malaria.ti,ab,kw. or dengue.ti,ab,kw. or rift valley fever.ti,ab,kw. or yellow fever.ti,ab,kw. or zika.ti,ab,kw. or japanese encephalitis.ti,ab,kw. or lymphatic filariasis.ti,ab,kw. or West Nile fever.ti,ab,kw. or Schistosomiasis.ti,ab,kw. or Sandfly fever.ti,ab,kw. or phelebotomus fever.ti,ab,kw. or bilharziasis.ti,ab,kw. or filariasis.ti,ab,kw. or chikungunya.ti,ab,kw. or leishmaniasis.ti,ab,kw. or Crimean-Congo haemorrhagic fever.ti,ab,kw. or Lyme disease.ti,ab,kw. or Relapsing fever.ti,ab,kw. or borreliosis.ti,ab,kw. or Rickettsial diseases.ti,ab,kw. or spotted fever.ti,ab,kw. or Q fever.ti,ab,kw. or Tick-borne encephalitis.ti,ab,kw. or Tularaemia.ti,ab,kw. or Chagas disease.ti,ab,kw. or American trypanosomiasis.ti,ab,kw. or Sleeping sickness.ti,ab,kw. or African trypanosomiasis.ti,ab,kw. or Plague.ti,ab,kw. or Rickettsiosis.ti,ab,kw. or Onchocerciasis.ti,ab,kw. or river blindness.ti,ab,kw. or Rabies.ti,ab,kw. or trachoma.ti,ab,kw. or buruli ulcer.ti,ab,kw. or endemic treponematoses.ti,ab,kw. or food-borne trematodiases.ti,ab,kw. or echinococcosis.ti,ab,kw. or leprosy.ti,ab,kw. or cysticercosis.ti,ab,kw. or tuberculosis.ti,ab,kw. or Filovirus diseases.ti,ab,kw. or ebola virus.ti,ab,kw. or marburg.ti,ab,kw. or lassa fever.ti,ab,kw. or Middle East Respiratory Syndrome.ti,ab,kw. or mers.ti,ab,kw. or Severe acute respiratory syndrome.ti,ab,kw. or sars.ti,ab,kw. or nipah.ti,ab,kw. or avian influenza.ti,ab,kw. or Coccidioides immitis.ti,ab,kw. or Coccidioides posadasii.ti,ab,kw. Or cholera.ti,ab,kw. (492 results)

AND

epidemic$.ti,ab,kw. or pandemic$.ti,ab,kw. or outbreak$.ti,ab,kw. or emergenc$.ti,ab,kw. or surge.ti,ab,kw. or re-occurrence.ti,ab,kw. or emergency relief.ti,ab,kw. or detection.ti,ab,kw. Or re?emergence.ti,ab,kw. (543 results)

AND

Containment measure$.ti,ab,kw. or control measure$.ti,ab,kw. or infection control.ti,ab,kw. or (control$ ADJ4 disease$).ti,ab,kw. or control$ strateg$.ti,ab,kw. or epidemic control.ti,ab,kw. or intervention measure$.ti,ab,kw. or control strateg$.ti,ab,kw. or control$.ti,ab,kw. or transmission containment.ti,ab,kw. or transmission control.ti,ab,kw. or (stop$ ADJ3 epidemic$).ti,ab,kw. or disease-control measure$.ti,ab,kw. or emergency response$.ti,ab,kw. or response intervention$.ti,ab,kw. or eliminat$.ti,ab,kw. or eradicat$.ti,ab,kw. or Emergency Preparedness.ti,ab,kw. or operational response$.ti,ab,kw. or mosquito$ control.ti,ab,kw. or interruption.ti,ab,kw. (6879 results)

Résultats combinés: 48

3.6 Search for OpenGrey

("Vector-borne disease*" OR "mosquito-borne disease*" OR "infectious NEAR/1 disease*" OR "emerging infectious disease*" OR "re-emerging infectious disease*" OR "communicable disease*" OR "emerging communicable disease*" OR "reemerging communicable disease*" OR "neglected tropical disease*" OR "disease Vector*" OR "Insect vector*" OR "arthropod vector*" OR "aracnid vector*" OR "tropical disease*" OR "borreliosis" OR "tularaemia" OR " american trypanosomiasis" OR "malaria" OR "dengue" OR " rift valley fever" OR "yellow fever" OR "zika" OR "japanese encephalitis" OR "lymphatic filariasis " OR "communicable disease*" OR "emerging communicable disease*" OR "reemerging communicable disease*" OR "neglected tropical disease*" OR "disease Vector*" OR "West Nile fever" OR "Schistosomiasis" OR "Sandfly fever" OR "phelebotomus fever" OR "bilharziasis" OR "filariasis" OR "chikungunya" OR "leishmaniasis" OR "Crimean-Congo haemorrhagic fever" OR "Lyme disease" OR "Relapsing fever" OR "borreliosis" OR "Rickettsial diseases" OR "spotted fever" OR "Q fever" OR "Tick-borne encephalitis" OR "Tularaemia" OR "Chagas disease" OR "American trypanosomiasis" OR "Sleeping sickness" OR "African trypanosomiasis" OR "Plague" OR "rickettsiosis" OR "Onchocerciasis" OR "river blindness" OR "Rabies" OR "trachoma" OR "buruli ulcer" OR "endemic treponematoses" OR "food-borne trematodiases" OR "echinococcosis" OR "leprosy" OR "cysticercosis" OR "tuberculosis" OR "filovirus diseases" OR "ebola virus" OR "marburg" OR "lassa fever" OR "Middle East Respiratory Syndrome" OR "mers" OR "Severe acute respiratory syndrome" OR "sars" OR "nipah" OR "avian influenza" OR "Coccidioides immitis" OR "Coccidioides posadasii" OR "cholera") AND (Urban$ OR "urban area" OR "urban population" OR "urban setting" OR "urban NEAR/2 health" OR "city" OR "metropolitan area" OR "metropolitan" OR "non?rural" OR "neighborhood" OR "downtown" OR "midtown" OR "uptown" OR "building" OR "district" OR "slum" OR "barrio" OR "township" OR "city center" OR "municipal$" OR "civic" OR "densely populated" OR "suburban" OR "town" OR "informal settlement") AND ("Containment measure*" OR "control measure*" OR "infection control" OR "control* NEAR/3 disease*" OR "control* strateg*" OR "epidemic control" OR "intervention measure*" OR "control strateg*" OR "control*" OR "transmission containment" OR "transmission control" OR "stop* NEAR/3 epidemic" OR "disease-control measure*" OR "emergency response*" OR "response intervention*" OR "Disease Control" OR "eliminat*" OR "eradicat*" )

Résultats: 37

("Vector-borne disease*" OR "mosquito-borne disease*" OR "infectious NEAR/1 disease*" OR "emerging infectious disease*" OR "re-emerging infectious disease*" OR "communicable disease*" OR "emerging communicable disease*" OR "reemerging communicable disease*" OR "neglected tropical disease*" OR "disease Vector*" OR "Insect vector*" OR "arthropod vector*" OR "aracnid vector*" OR "tropical disease*" OR "borreliosis" OR "tularaemia" OR " american trypanosomiasis" OR "malaria" OR "dengue" OR " rift valley fever" OR "yellow fever" OR "zika" OR "japanese encephalitis" OR "lymphatic filariasis " OR "communicable disease*" OR "emerging communicable disease*" OR "reemerging communicable disease*" OR "neglected tropical disease*" OR "disease Vector*" OR "West Nile fever" OR "Schistosomiasis" OR "Sandfly fever" OR "phelebotomus fever" OR "bilharziasis" OR "filariasis" OR "chikungunya" OR "leishmaniasis" OR "Crimean-Congo haemorrhagic fever" OR "Lyme disease" OR "Relapsing fever" OR "borreliosis" OR "Rickettsial diseases" OR "spotted fever" OR "Q fever" OR "Tick-borne encephalitis" OR "Tularaemia" OR "Chagas disease" OR "American trypanosomiasis" OR "Sleeping sickness" OR "African trypanosomiasis" OR "Plague" OR "rickettsiosis" OR "Onchocerciasis" OR "river blindness" OR "Rabies" OR "trachoma" OR "buruli ulcer" OR "endemic treponematoses" OR "food-borne trematodiases" OR "echinococcosis" OR "leprosy" OR "cysticercosis" OR "tuberculosis" OR "filovirus diseases" OR "ebola virus" OR "marburg" OR "lassa fever" OR "Middle East Respiratory Syndrome" OR "mers" OR "Severe acute respiratory syndrome" OR "sars" OR "nipah" OR "avian influenza" OR "Coccidioides immitis" OR "Coccidioides posadasii") AND ("epidemic*" OR "pandemic*" OR "outbreak*" OR "emergenc*" OR "surge" OR "re-occurrence" OR "emergency relief" OR "detection")

Résultats: 221

3.7 Search for WHOLIS

Subject "vector-borne diseases" OR subject "infectious diseases" OR subject "communicable diseases" AND subject "epidemic" OR subject "emergency" AND subject "control" OR subject "containment"

Résultats: 189

subject "disease control" AND subject "communicable diseases" OR subject "vector-borne diseases"

Résultats: 461

Records identified through database searching: 5822

Records after duplicates removed : 4192
